# Retinal Phenotyping of a Murine Model of Lafora Disease

**DOI:** 10.3390/genes14040854

**Published:** 2023-03-31

**Authors:** Ajoy Vincent, Kashif Ahmed, Rowaida Hussein, Zorana Berberovic, Anupreet Tumber, Xiaochu Zhao, Berge A. Minassian

**Affiliations:** 1 Department of Ophthalmology and Vision Sciences, Hospital for Sick Children, Toronto, ON M5G 1X8, Canada; 2 Genetics and Genome Biology, Hospital for Sick Children, Toronto, ON M5G 0A4, Canada; 3 Department of Ophthalmology and Vision Sciences, University of Toronto, Toronto, ON M5T 3A9, Canada; 4 The Center for Phenogenomics, Toronto, ON M5T 3H7, Canada; 5 Division of Neurology, Department of Pediatrics, University of Texas Southwestern Medical Center, Dallas, TX 75390, USA

**Keywords:** disease, Lafora body disorder, Lafora, *EPM2A*, electroretinography, optical coherence tomography, periodic acid Schiff reaction, phenotype, knock outs, gene

## Abstract

Lafora disease (LD) is a progressive neurologic disorder caused by biallelic pathogenic variants in *EPM2A* or *EPM2B*, leading to tissue accumulation of polyglucosan aggregates termed Lafora bodies (LBs). This study aimed to characterize the retinal phenotype in *Epm2a^−/−^* mice by examining knockout (KO; *Epm2a^−/−^*) and control (WT) littermates at two time points (10 and 14 months, respectively). In vivo exams included electroretinogram (ERG) testing, optical coherence tomography (OCT) and retinal photography. Ex vivo retinal testing included Periodic acid Schiff Diastase (PASD) staining, followed by imaging to assess and quantify LB deposition. There was no significant difference in any dark-adapted or light-adapted ERG parameters between KO and WT mice. The total retinal thickness was comparable between the groups and the retinal appearance was normal in both groups. On PASD staining, LBs were observed in KO mice within the inner and outer plexiform layers and in the inner nuclear layer. The average number of LBs within the inner plexiform layer in KO mice were 1743 ± 533 and 2615 ± 915 per mm^2^, at 10 and 14 months, respectively. This is the first study to characterize the retinal phenotype in an *Epm2a^−/−^* mouse model, demonstrating significant LB deposition in the bipolar cell nuclear layer and its synapses. This finding may be used to monitor the efficacy of experimental treatments in mouse models.

## 1. Introduction

Lafora Disease (LD), first described in 1911 [1], is a rare progressive myoclonus epilepsy due to biallelic pathogenic variants in *EPM2A* or *EPM2B* [2,3,4]. The disease typically manifests in adolescence with focal visual seizures or visual hallucinations [5]. This is soon followed by action and stimulus-sensitive myoclonus, as well as tonic-clonic and absence seizures [6,7]. Neuro-psychiatric symptoms that include behavioural changes, depression and apathy are also often seen early in the disease [5,6,7]. These initial symptoms rapidly deteriorate, and patients develop progressive dementia, psychosis, cerebellar ataxia, dysarthria, muscle wasting and respiratory failure that invariably leads to death within a decade [4,5,6,7,8]. In early disease, electroencephalography (EEG) typically shows spikes and polyspikes on a slowed background, both when awake and during sleep [6,9,10,11,12]. As the disease progresses, EEG demonstrates long bursts of diffuse spike-waves and fast polyspikes associated with myoclonic jerks and enhanced by photic stimulation [6,9,10,11,12]. Although, subjects with LD typically retain good visual acuity and color vision [13,14], retinal structure and function are often abnormal [13,14,15]. For instance, on full-field electroretinogram (ERG) testing, a generalized cone dysfunction is commonly observed [13,14]; additional involvement of the rod bipolar system may be seen in some patients [13]. Optical coherence tomography (OCT), an in vivo imaging technique, shows preservation of retinal lamination [13], but regional thinning is described in some patients [15]. Further, adaptive optics scanning light ophthalmoscopy (AOSLO), a non-invasive technique capable of resolving single-cell structures, shows nummular hyperreflective structures within the retinal nerve fiber layer in LD [15].

Lafora Disease has been reported in multiple dog breeds [16,17,18,19,20] as an autosomal recessive trait caused by repeat expansion variation in *NHLRC1 (EPM2B)* [19,20,21,22], with an average age of disease onset of 7 years [18,19,23,24]. The common manifestations in dogs include spontaneous and reflex myoclonus, hypnic myoclonus, generalized tonic-clonic and focal seizures, and jaw smacking [24]. The disease symptoms progressively intensify, and dogs are typically euthanized in late disease [20,24,25]. The EEG findings in dogs are similar to human LD and include spontaneous or visually evoked solitary spikes, bursts or polyspikes, with the latter being easily evoked by photic stimulation [21,26]. Impaired vision or blindness has been described in about 40% of affected dogs in late disease stages and is uncommon in early disease [24]; however, the cause for vision impairment is currently unknown. Knockout mouse models of *Epm2a^−/−^* and *Epm2b^−/−^* have been generated [27,28,29]. The *Epm2a^−/−^* mice developed behavioral abnormalities at 4 months of age, motor coordination and memory deficits between 4 and 8 months, and myoclonus by 9 months [27,30]; these deficits worsen over time [30]. Older *Epm2a^−/−^* mice showed spike and slow-wave formation on EEG; notably, the regular and well-formed diffuse 3–6 Hz spike-wave complexes found in human LD are absent in this mouse model even at 1 year of age [27]. However, one study reported epileptiform activity in *Epm2a^−/−^* mice as young as 3 months [30]. The *Epm2b^−/−^* mice developed impaired motor coordination, memory deficits and epileptiform activity including tonic-clonic seizures between 3 and 8 months of age that progressively worsened [30,31]; however, a different strain of *Epm2b^−/−^* showed no behavioural, cognitive or learning abnormalities up to 11 months of age, suggesting variability between genetic strains [29]. The EEG of these mice showed spontaneous slow spike, spike-wave, and poly spike-wave complexes with or without corresponding myoclonic jerks [30].

A pathological hallmark of LD is the progressive deposition of polyglucosan aggregates in tissues, termed Lafora Bodies (LBs) [1]. In humans, LBs have been identified in the brain, liver, skeletal and cardiac muscle, sweat glands and retina [32,33,34,35,36,37]. In canine LD, LBs have been identified in the brain, liver, apocrine and eccrine sweat glands, and muscle tissue [20,23,25,38]. In *Epm2a* and *Epm2b* knock-out (KO) mice, LBs were identified in the various regions of the brain, footpads, as well as skeletal and cardiac muscles [27,28].

Currently, multiple therapeutic options are being pursued for LD [39]. Since the retina can be easily assessed to monitor treatment efficacy, identification of a retinal phenotype in an animal model of LD could accelerate the development of such therapies. Hence, the current study, aimed to ascertain and characterize the retinal phenotype in a KO model of Lafora disease (*Epm2a^−/−^*) by performing in-depth in vivo and ex vivo assessments.

## 2. Materials and Methods

### 2.1. Mouse Line

The *Epm2a^−/−^* KO mice were a gift from Drs. Subramaniam Ganesh and Antonio Delgado–Escueta. Briefly, the mice were generated by deleting the dual-specificity phosphatase domain (DPSD) coding region (exon 4) of the *Epm2a* gene and replacing it with a neomycin resistance gene via homologous recombination [27]. The absence of gene expression was verified by the Northern blot and RT-PCR analysis of brain and liver tissue from homozygous mutant animals using probes to the DSPD and upstream exons 1–3 [27]. Mice were housed in individually ventilated cages at 20 to 22 °C with access to water and food. Animal procedures were approved by The Centre for Phenogenomics Animal Care Committee and in compliance with the Canadian Council for Animal Care Guidelines.

### 2.2. Electroretinogram Testing, Retinal Photography and Optical Coherence Tomography

Five knockouts (KO; *Epm2a^−/−^*) and control (WT) littermates were examined at two time points (10 and 14 months, respectively). After overnight dark adaptation (DA), pupils were dilated using tropicamide 1% and phenylephrine 2.5% eye drops. Subsequently, mice were injected with 0.01 mL/g body weight of anesthetic solution (combination of 100 mg/mL Ketamine and 20 mg/mL Xylazine). Binocular electroretinogram (ERG) testing was then performed using the Lab Cradle (Diagnosys LLC) system. A DA (scotopic) intensity series ERG was performed (9 steps; 0.0025–10 cd·s·m^−2^) followed by a 10-min light adaptation (LA; 30 cd.m^−2^). Subsequently, LA ERGs were performed using a 5 cd·s·m^−2^ stimulus flash to four stimulus frequencies (5, 10, 15 and 20 Hz). For analysis, the ERG from the eye with superior technical quality was chosen. If responses from either eye were similar, the right eye was chosen. Both a-wave and b-wave amplitudes were measured for the steps of the DA ERG. The a-wave amplitude was measured from the baseline to the trough of the first negative waveform, whilst the b-wave amplitude was measured from the trough of the a-wave to the peak of the subsequent positive waveform. The a-wave amplitudes were measured for 7 light intensities (DA 0.01 cd·s·m^−2^ to DA 10 cd·s·m^−2^) and the b-wave amplitudes were measured for all steps of the DA ERG. For the LA ERG, the trough-to-peak amplitude of the responses was calculated for all four stimulus frequencies.

**Figure 1:**
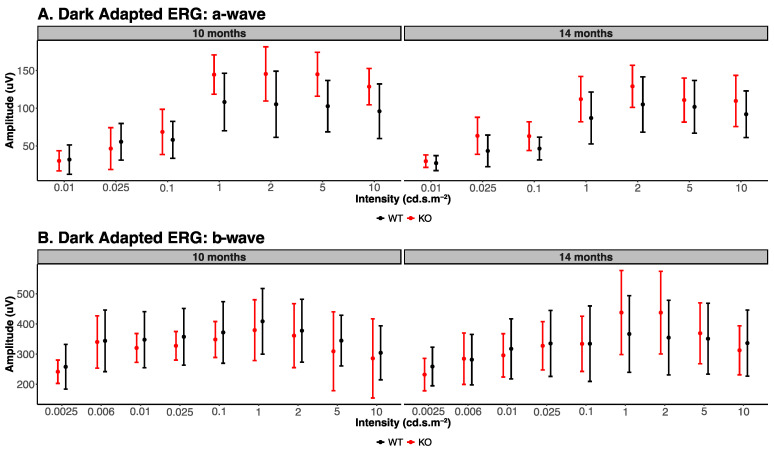

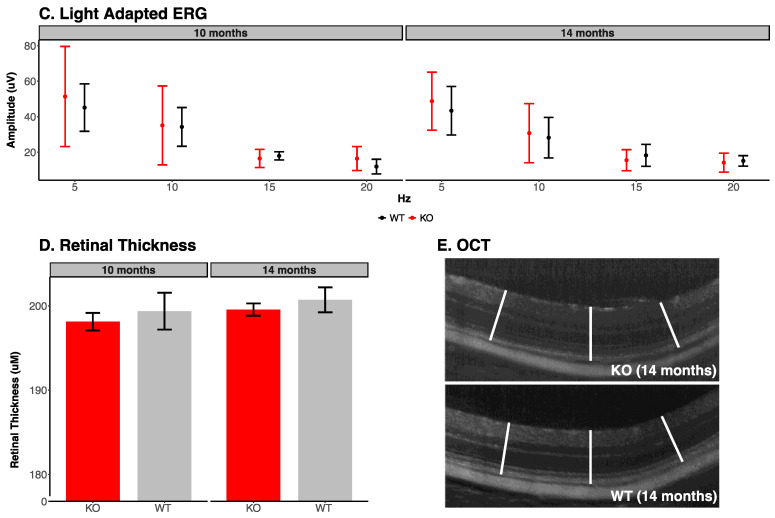
Full-field electroretinogram (ERG) and optical coherence tomography (OCT) findings in Epm2a^−/−^ knock out (KO) mice and control (WT) littermates. (**A**) Dark-adapted (DA) ERG a-wave amplitude measures to different intensities of flash (ranging from 0.01 cd·s·m^−2^ to 10 cd·s·m^−2^) showed no difference between KO and WT mice at both 10 months and 14 months of age. (**B**) The DA ERG b-wave amplitude measures to different intensities of flash (ranging from 0.0025 cd·s·m^−2^ to 10 cd·s·m^−2^) showed no difference between KO and WT mice at both time points. (**C**) Light-adapted (LA) ERG amplitude measures to four different flicker frequencies (5–20 Hz) showed no difference between KO and WT mice at both time points. Notably, the LA flicker amplitudes are lower at higher stimulus frequencies (15 and 20 Hz) compared to lower stimulus frequencies (5 and 10 Hz) in both KO and WT mice. This is because the depolarizing bipolar cell contribution that predominates the LA ERG at lower flicker frequencies shows larger amplitudes compared to the hyperpolarizing bipolar cell contribution that predominates at high flicker frequencies. Similar findings have been consistently observed in multiple WT mouse models. (**D**) The average retinal thickness was comparable between KO and WT mice at both time points. (**E**) A representative horizontal line scan from KO and WT mice, respectively, showing how the average retinal thickness was calculated.

After ERG testing, fundus imaging and retinal OCT were performed using Phoenix MICRON-III™. Retinal photographs were obtained from either eye to ascertain any visible retinal abnormalities. For OCT, horizontal single line scans (1.8 mm diameter, 1.02 µm depth) were performed centered at the optic disc and at regions dorsal and ventral to the disc in each eye. The OCT allows for the detection of changes to the retinal thickness and lamination; also, the OCT can detect abnormal retinal deposition or any disruption to the retinal layers [40,41,42,43]. For quantifying retinal thickness, a horizontal line scan from the right eye, close to the optic disc was chosen in each mouse. Next, using ImageJ (Version 1.53t), the distance between the retinal pigment epithelium and the internal limiting membrane was manually calculated from three different locations from within the scan (Figure 1E) and was averaged to obtain the average retinal thickness in each mouse.

### 2.3. Histological Analysis

After the ERG and OCT testing, animals were sacrificed by cervical dislocation. Eyes were removed and immediately fixed in 10% buffered formalin followed by paraffin embedding. Paraffin-embedded retinal tissue was sectioned and stained with the Periodic acid Schiff-Diastase (PASD) at The Centre for Phenogenomics Pathology Services, Toronto. PASD is a sensitive histochemical method to assess for glycogen; diastase application depolymerises glycogen to maltose and glucose, which are washed out of the slide [44]. The LBs are polyglucosan aggregates resistant to diastase and as such are easily identifiable after PASD staining [6,45,46]. After PASD staining, slides were imaged at 40× using 3DHistech Pannoramic Flash II Slide Scanner at the Hospital for Sick Children Imaging facility, Toronto. Slides were analyzed using 3DHistech CaseViewer software and LBs were counted using 3DHistech QuantCenter. Briefly, regions of interest were selected on the retina sections, and a scenario was built in QuantCentre that distinguishes LBs from the background and other structures within the inner plexiform layer (IPL). The same scenario was used for both WT and KO samples to count LBs.

## 3. Results

### 3.1. Electroretinogram Analysis

Full-field ERG was performed to assess the effects of LBs deposition on retinal function. Table 1 and Figure 1A–C details the summary of the ERG findings. Briefly, there was no significant difference seen for any of the a- or b-wave amplitude measures of the DA ERG, between KO and WT mice, at either time point. Similarly, LA ERG amplitudes showed no significant difference between KO and WT mice at both time points.

### 3.2. Retinal Photography and Optical Coherence Tomography

Both KO and WT mice had a normal appearance on retinal photography at both 10 and 14 months. Overall, on OCT imaging, the retinal lamination appeared preserved and no localized changes in reflectivity were observed in KO mice. The average retinal thickness in KO mice was 198.53 ± 0.97 µm and 199.86 ± 0.67 µm at 10 and 14 months, respectively (Figure 1D). The average retinal thickness in WT mice was 199.69 ± 2.02 µm and 200.93 ± 1.37 µm at 10 months and 14 months, respectively (Figure 1D). These results show that average retinal thickness was similar in both KO and WT groups, at both 10 (*p* = 0.29) and 14 months (*p* = 0.17).

### 3.3. Periodic Acid Schiff-Diastase Staining of the Retina

**Figure 2:**
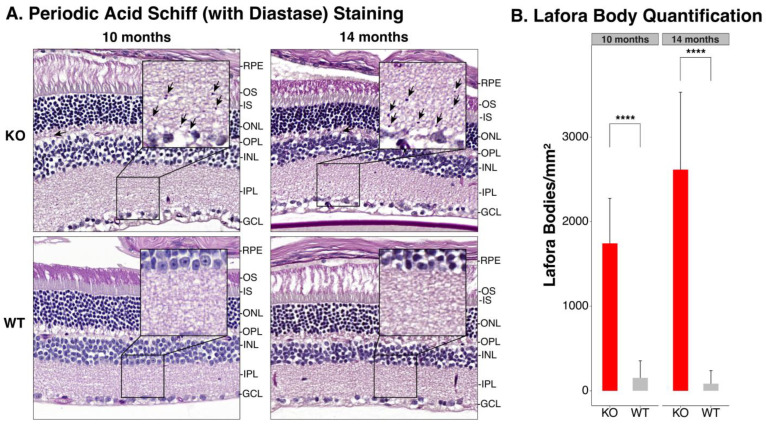
Periodic Acid Schiff-Diastase) (PASD) staining in *Epm2a^−/−^* knock out (KO) mice and control (WT) littermates. (**A**) PASD staining of KO mice shows Lafora body (LB) deposition in KO mice at both 10 and 14 months (**top** panel). The LBs (black arrows) were noted in the inner plexiform layer (IPL), inner nuclear layer (INL) and outer plexiform layer (OPL). The LBs were most numerous in the IPL. The LBs were not visualised in WT littermates (**bottom** panel). All the other retinal layers are also labelled: retinal pigmented epithelium (RPE), photoreceptor outer segment (OS), photoreceptor inner segment (IS), outer nuclear layer (ONL) and ganglion cell layer (GCL). (**B**) Automated quantification of the LBs within the IPL showed a significantly higher LB deposition in KO mice compared to WT mice at both time points (**** *p* < 0.0001). There is a significant increase in the number of LBs in KO mice at 14 months, in comparison to 10 months (*p* = 0.035).

Periodic acid Schiff-Diastase staining was executed to label LBs in the retinal tissue. The LBs were only observed in the retinas of KO mice and not in the WT littermates (Figure 2A, upper panels black arrows). In the KO mice retina, numerous LBs were observed within the IPL; LBs were also present less frequently in the inner nuclear layer (INL) and the outer plexiform layer (OPL). Upon quantification of LBs in the IPL, the average number of LB accumulation in KO mice was 1743 ± 533 and 2615 ± 915 per mm^2^ at 10 and 14 months, respectively (Figure 2B). There were no identifiable LBs in the IPL of WT mice (Figure 2A, lower panels), but the software identified some of the background noise in slides as LBs, which numbered 153 ± 202 and 84 ± 154 per mm^2^ at 10 and 14 months, respectively. There was a statistically significant difference in the amount of LB deposition between KO and WT mice at both 10 (*p* < 0.0001) and 14 months (*p* < 0.0001). There was a significant increase in LB deposition over time in KO mice (*p* = 0.035). Further, the average diameter of the LB in KO mice was 0.45 ± 0.11 µm (range: 0.31–0.60 µm) and 0.47 ± 0.14 µm (range: 0.30–0.71 µm) at 10 and 14 months, respectively.

## 4. Discussion

This *Epm2a^−/−^* mouse model demonstrates LB deposition in the inner and outer plexiform layers of the retina, as well as in the INL. The LBs were most numerous in the IPL which represents the synapse of the bipolar cell neuron with the ganglion cell neuron. Intriguingly, in microscopic studies of human LD [36], LBs were identified in the same layers as our *Epm2a^−/−^* mouse model. This novel finding indicates some reminiscence of this *Epm2a^−/−^* mouse model to human LD. Further, retinal photographs in the *Epm2a^−/−^* mice in the current study were normal, and similar to WT littermates. Human patients with molecularly confirmed LD also demonstrate normal retinal appearance in color photography [13,14].

In the current study, full-field ERG was performed to assess the generalized retinal function. The a- and b-waves of the DA mouse ERG arise from the rod photoreceptors and bipolar cells, respectively [47,48,49,50]. The mouse LA ERG has its origins from the cone photoreceptors, and cone depolarizing and hyperpolarizing bipolar cells; whilst the depolarizing bipolar cell contribution is higher between 5 and 15 Hz stimulus frequencies, the hyperpolarizing bipolar cell contribution predominates at 20 Hz stimulus frequency [51,52,53,54,55]. All tested ERG parameters were normal in KO mice, and none of the parameters showed any difference from WT littermates. The normal DA a-wave amplitudes may be explained by the absence of LB deposition in the photoceptor layer on PASD staining. Although there was some degree of LB deposition in the INL, DA b-wave amplitude and LA flicker amplitudes were also normal; these results may indicate that there is not enough LB deposition in the inner retina to cause a detectable dysfunction. On the contrary, human patients with LD consistently show ERG abnormalities [13,14], and perhaps, this represents a distinction in the degree of retinal involvement between human LD and this *Epm2a^−/−^* mouse model. Notably, there are also differences in the degree and timing of the neurological and behavioural deficits observed amongst various LD mouse models themselves [27,29,30,31], as well as between mouse models and human LD [27,30]. It is likely that other factors including environmental, genetic or species-specific may contribute to the observed differences in phenotypes [27,30]. Further, *Epm2a* and *Epm2b* that encode laforin and malin, respectively, are thought to function as a complex, and KO mice demonstrate defects in autophagy and protein clearance [31,56,57,58,59,60,61]. That said, there is also evidence to suggest that malin may have roles independent of laforin [31,56]. Hence, *Epm2a* and *Epm2b* double knockout mice could be a better model of LD and perhaps, show more pronounced retinal dysfunction compared to the *Epm2a^−/−^* mice in the present study.

The retinal lamination was preserved in KO mice at both time points. This finding is similar to human LD, where also, the retinal lamination is preserved [13,14]. The average retinal thickness in KO mice was similar to WT littermates at both 10 months and 14 months. In human LD, there is a single study, which reported regional inner retinal thinning within the macula in two patients [15]. A conference abstract on *Epm2b^−/−^* mice (in 2018) also noted thinning of the retinal nerve fiber layer [62], although no further details were available. The mouse retina lacks a macula and the MICRON-III system used to image *Epm2a^−/−^* mice in the current study did not have the capability to measure regional retinal thickness, limiting our analysis. Whilst the LBs were seen on PASD staining in the KO mice, they were not visualised on in vivo OCT scanning. This is likely due to the average diameter of LB in KO mice measuring ~0.45 and 0.47 µm at 10 and 14 months, respectively, which is beyond the axial resolution capability of the OCT system (~3 µm). Upon review of the literature, the size of LB noted in the hippocampus in *Epm2a^−/−^* mice at 9 months appeared to be ≤2 µm [27] and those identified in the human retina ranged between 2–10 µm [36]. Hence, to be able to in vivo image LB deposition in the retina of *Epm2a^−/−^* mice, one might need higher resolution OCT or AOSLO technology (<2 µm) [63]. It is notable, that a prior human study was able to identify Gunn’s dots (a biomarker for neurodegeneration) in the retinal nerve fiber layer using AOSLO imaging but was not successful in imaging LB [15]. Further, in our study, a notable increase in retinal LB deposition was observed at 14 months, compared to 10 months.

Since the primary aim of our study was to characterize the eye phenotype in *Epm2a^−/−^* mice, we did not ascertain LB deposition in the brain, liver or skeletal muscle in these mice. In retrospect, this is a missed opportunity as we could have compared the increase in LBs in KO mice retina over time to any changes in LB accumulation over time in other tissues. It is noted that an increase in LB deposition in the brain over time (4 to 17 months of age) has previously been reported by others in the same *Epm2a^−/−^* mouse model [27,30].

To summarize, we have characterised the retinal phenotype in a KO mouse model of *Epm2a*, widely used as a human disease model for LD. Whilst these *Epm2a^−/−^* mice had deposition of LBs, a pathognomonic feature of LD, within the middle and inner retinal layers, these bodies did not lead to any structural and functional retinal defects that were identifiable in vivo. The degree of retinal involvement in this mouse model is milder than in the patients affected with *EPM2A*-related LD, as most human subjects showed ERG changes indicative of a functional defect. Nonetheless, as several treatment options are being explored for LD, the extent of LB deposition in the retina may be monitored ex vivo in the *Epm2a^−/−^* mice to ascertain treatment success for eye involvement. Further, in vivo monitoring of the neurological and behavioural deficits, as well as the EEG abnormalities in *Epm2a^−/−^* mice [27,30] could help ascertain treatment success for systemic features. Imaging techniques with higher lateral and axial resolution will likely enable visualization of LBs in vivo in the retina and could be the focus of future studies.

## Figures and Tables

**Table 1:** ERG Parameters in *Epm2a* knockout and wildtype mice.

Dark Adapted ERG Parameters (10 Months)						
Stimulus Flash in cd·s·m^−2^	A Wave			B Wave		
	*Knockout*	*Wildtype*	*p-Value*	*Knockout*	*Wildtype*	*p-Value*
0.0025	-	-		242 ± 38.76	258 ± 74.16	0.68
0.006	-	-		340 ± 86.56	344 ± 102.19	0.95
0.01	27 ± 9.91	32 ± 19.47	0.62	320 ± 47.77	348 ± 93.18	0.57
0.025	43 ± 20.98	55 ± 24.24	0.47	328 ± 47.30	357 ± 94.02	0.55
0.1	46 ± 15.13	58 ± 24.46	0.38	348 ± 59.59	372 ± 102.17	0.66
1	87 ± 34.40	108 ± 38.10	0.39	379 ± 100.68	409 ± 109.05	0.66
2	105 ± 36.65	105 ± 43.86	1.00	361 ± 106.10	378 ± 104.35	0.80
5	102 ± 34.97	103 ± 34.08	0.96	309 ± 130.79	345 ± 83.99	0.62
10	92 ± 30.89	96 ± 36.24	0.86	286 ± 131.38	304 ± 89.58	0.81
**Dark Adapted ERG Parameters (14 Months)**						
**Stimulus Flash in cd·s·m^−2^**	**A Wave**			**B Wave**		
	***Knockout***	***Wildtype***	***p-Value***	***Knockout***	***Wildtype***	***p-Value***
0.0025	-	-		232 ± 53.71	259 ± 64.01	0.49
0.006	-	-		285 ± 85.24	282 ± 83.61	0.96
0.01	30 ± 8.29	30 ± 13.33	1.00	296 ± 71.82	317 ± 99.56	0.71
0.025	63 ± 24.55	46 ± 27.68	0.33	328 ± 80.20	335 ± 109.26	0.91
0.1	63 ± 19.07	69 ± 30.05	0.72	334 ± 91.61	334 ± 125.15	1.00
1	112 ± 30.08	145 ± 26.07	0.10	438 ± 139.30	367 ± 127.21	0.42
2	129 ± 27.96	145 ± 35.95	0.45	437 ± 136.87	355 ± 123.90	0.35
5	111 ± 29.22	145 ± 29.12	0.10	369 ± 101.02	351 ± 117.61	0.80
10	110 ± 33.93	129 ± 24.05	0.34	313 ± 81.42	337 ± 109.48	0.70
**Light Adapted ERG Parameters (10 Months)**						
**Stimulus Frequency in Hz**	**Trough to Peak Amplitude**					
	***Knockout***		***Wildtype***			***p-Value***
5	49.98 ± 25.47		47.93 ± 13.78			0.89
10	37.25 ± 20.57		36.72 ± 11.47			0.97
15	17.05 ± 4.79		18.27 ± 2.22			0.62
20	16.99 ± 6.21		12.9 ± 4.46			0.27
**Light Adapted ERG Parameters (14 Months)**						
**Stimulus Frequency in Hz**	**Trough to Peak Amplitude**					
	***Knockout***		***Wildtype***			***p-Value***
5	48.74 ± 16.32		43.38 ± 13.65			0.59
10	30.7 ± 16.67		28.18 ± 11.45			0.79
15	15.5 ± 5.94		18.20 ± 6.22			0.50
20	14.1 ± 5.37		15.09 ± 2.96			0.73

## Data Availability

The data presented in this study are available on request from the corresponding author.
